# Enabling Navigation and Augmented Reality in the Sitting Position in Posterior Fossa Surgery Using Intraoperative Ultrasound

**DOI:** 10.3390/cancers16111985

**Published:** 2024-05-23

**Authors:** Miriam H. A. Bopp, Alexander Grote, Marko Gjorgjevski, Mirza Pojskic, Benjamin Saß, Christopher Nimsky

**Affiliations:** 1 Department of Neurosurgery, University of Marburg, Baldingerstrasse, 35043 Marburg, Germany; alexander.grote@uk-gm.de (A.G.); marko.gjorgjevski@uk-gm.de (M.G.); mirza.pojskic@uk-gm.de (M.P.); sassb@med.uni-marburg.de (B.S.); nimsky@med.uni-marburg.de (C.N.); 2 Center for Mind, Brain and Behavior (CMBB), 35043 Marburg, Germany

**Keywords:** posterior fossa, cerebellar tumor, sitting, neuronavigation, augmented reality, navigation update, ultrasound, intraoperative ultrasound, accuracy

## Abstract

**Simple Summary:**

Neuronavigation and microscope-based augmented reality are widely used in neurosurgery to support intraoperative orientation, preserve neurological function, and maximize the extent of resection. However, in the sitting position, navigation may not be accurate enough to fully exploit its potential due to brain deformations caused by gravity and brain shift. To ensure accurate navigation and augmented reality support, it is necessary to verify and update navigation regularly. Intraoperative ultrasound is an easy-to-use tool that can be used to verify accuracy and to generate real-time image data to update navigation without significantly interrupting the surgical workflow. This can be achieved by outlining the lesion within the data set or by rigidly co-registering preoperative and intraoperative data to update and enable navigation and augmented reality support. In this study, image-based co-registration improved the navigation accuracy, making intraoperative ultrasound useful for enabling navigation and augmented reality support during posterior fossa surgery in the sitting position.

**Abstract:**

Despite its broad use in cranial and spinal surgery, navigation support and microscope-based augmented reality (AR) have not yet found their way into posterior fossa surgery in the sitting position. While this position offers surgical benefits, navigation accuracy and thereof the use of navigation itself seems limited. Intraoperative ultrasound (iUS) can be applied at any time during surgery, delivering real-time images that can be used for accuracy verification and navigation updates. Within this study, its applicability in the sitting position was assessed. Data from 15 patients with lesions within the posterior fossa who underwent magnetic resonance imaging (MRI)-based navigation-supported surgery in the sitting position were retrospectively analyzed using the standard reference array and new rigid image-based MRI-iUS co-registration. The navigation accuracy was evaluated based on the spatial overlap of the outlined lesions and the distance between the corresponding landmarks in both data sets, respectively. Image-based co-registration significantly improved (*p* < 0.001) the spatial overlap of the outlined lesion (0.42 ± 0.30 vs. 0.65 ± 0.23) and significantly reduced (*p* < 0.001) the distance between the corresponding landmarks (8.69 ± 6.23 mm vs. 3.19 ± 2.73 mm), allowing for the sufficient use of navigation and AR support. Navigated iUS can therefore serve as an easy-to-use tool to enable navigation support for posterior fossa surgery in the sitting position.

## 1. Introduction

With its introduction in the 1990s, image-guided surgery and neuronavigation have become indispensable tools for many cranial and spinal surgical procedures [1,2]. Already proving its clinical usefulness and benefits in identifying deep-seated lesions, precisely defining resection margins, and preserving functional risk structures, imaging-based neuronavigation has broadly become a routine intrinsic part of surgical procedures [3,4]. The implementation of microscope-based augmented reality (AR), which allows for real-time AR visualization of outlined lesions and risk structures, thus supporting the surgeon’s mental transfer of relevant information between the image space and the surgical field, reducing the need for attention shifts and increasing surgeon comfort, further complements navigation-assisted intraoperative guidance [5,6,7,8,9]. Despite the well-known benefits, neuronavigation and, thereby, microscope-based AR have not found their way into surgery within the posterior fossa on a routine basis.

Lesions within the cerebellum and brain stem account for approximately 5% of all brain tumors in adults and exhibit a rate of about 50% in children, mostly requiring resection or at least a biopsy for diagnostics and tailored treatment [10]. It is imperative to choose the best surgical approach with an optimal surgical trajectory to target the lesion while limiting tissue dissection to a minimum [11]. The usefulness of neuronavigation assistance, especially in the sitting position for posterior fossa surgery, has been doubted due to concerns of accuracy being impaired by gravitational effects and brain shift caused by cerebrospinal fluid (CSF) loss [11], which, besides the reduction in cerebellar swelling, the reduced surgical time and blood loss and the gravitational loss of venous blood are undisputable advantages of the sitting position for posterior fossa surgery [12,13] over surgery in, e.g., the prone position. As recently demonstrated, surgical positioning differing from patient positioning during preoperative magnetic resonance imaging (MRI) data acquisition also affects the clinical accuracy from the beginning of the surgery, leading to further inaccuracies in the prone and sitting position when accessing the posterior fossa [10,12,14]. 

Even though the overall navigation accuracy can be improved in different ways, enabling an optimized initial patient-to-image registration by, e.g., reducing effects due to skin shift and the proper placement of artificial registration landmarks, accuracy is known to constantly decrease during surgery and, in combination with the non-linear effects of brain shift, could, in the worst case, lead to an unacceptable mismatch of image and patient data [3,4,15]. While the positional shift of the patient’s head in relation to the reference array alone can be compensated for by rigid re-registration techniques using intraoperatively acquired landmarks, intracranial positional and intraparenchymal shifting can be addressed by intraoperative imaging techniques such as intraoperative MRI (iMRI) or intraoperative ultrasound (iUS), if applicable, or partially also by AR-based navigation updates [15]. Navigation updates using AR can compensate for in-plane inaccuracies but cannot account for inaccuracies along the focal axis of the operating microscope. The usage of iMRI is generally limited due to its availability, high costs, structural requirements, relevant interruption of the surgical procedure and time consumption, constraints in patient positioning, and partially not allowing for its application [16,17]. Contrarily, iUS can be performed at any time during surgery, also repeatedly, with no significant interruption of the surgical procedure, is widely available, cost-effective, and straightforward to use [18]. Despite its presumed lower imaging quality compared to classical MRI techniques, its user dependency, and a lack of training and experience in interpreting iUS data, with its integration into neuronavigation systems, iUS has gained more attraction and is now part of the surgical routine in many setups [19,20,21,22,23,24,25]. Navigated iUS allows for a regular exploration of the navigation accuracy, brain shift, and extent of resection depending on the echogenicity of the lesion. It also offers an opportunity to update navigation using intraoperatively acquired 3D iUS data. However, its potential might not yet be fully exploited.

According to the experience of our study group in intraoperative US application in cerebral metastasis [25], glioma surgery [20,21], and spine surgery [26], navigated iUS is now fully integrated into the workflow of most neurosurgical procedures. Therefore, first, navigated iUS imaging is typically performed, and an iUS data set is acquired before the dural opening. If needed, and up to the surgeon’s intraoperative impression, iUS can be applied at any time during the surgical procedure to verify the navigation accuracy or update navigation, depending on the lesion, to determine the extent of resection, or to identify the remaining tumor intraoperatively, leading to continued resection.

Considering the benefits of neuronavigation and the limitations of the navigation accuracy concerning positional effects, overall accuracy, and brain shift, the application of intraoperative navigation US and the acquisition of iUS data sets to evaluate navigation accuracy and compensate for inaccuracies seems to be an ideal and easy-to-use tool to enable neuronavigation even in posterior fossa surgery. This study therefore aims to assess the applicability and usefulness of iUS in neuronavigation-supported posterior fossa surgery to compensate for navigation inaccuracies.

## 2. Materials and Methods

### 2.1. Study Cohort

Data from 15 patients (male/female: 6/9, mean age: 60.27 ± 9.33 years) who consecutively underwent neuronavigation-supported microsurgical resection of suspected cerebellar lesions or lesions of the brainstem or within the fourth ventricle in a sitting approach after exclusion of patent foramen ovale (PFO) were retrospectively analyzed within this study. All surgeries were performed by a single surgeon (C.N.) with over 25 years of experience in intraoperative imaging to reduce effects of user dependency during intraoperative US usage. Patients with lesions close to bony structures, such as cerebellopontine angle tumors, were excluded in this proof-of-concept study as bone-related artifacts and the induced signal loss led to an incomplete depiction of the lesion in the iUS data, inhibiting a lesion-based analysis (spatial overlap of outlined lesions in MRI and iUS data). Ethics approval for prospectively archiving and collecting routine clinical and technical data during neurosurgical treatment of patients was obtained in accordance with the Declaration of Helsinki and was approved by the ethics committee at the University of Marburg (No. 99/18); analysis of these data within this study was additionally approved by the ethics committee (RS 23/10). All included patients provided written informed consent before participation.

### 2.2. Technical Equipment

Surgical planning and, later on, retrospective analysis of all included cases was performed on a dedicated planning server (Origin Server, Brainlab, Munich, Germany) equipped with various software modules (Brainlab Elements, Brainlab, Munich, Germany) including, among other things, software tools for image fusion, tumor segmentation, and definition of landmarks. 

For navigation-assisted surgery, both available neurosurgical operating rooms (ORs) are equipped with a Curve Navigation System (Brainlab, Munich, Germany), enabling patient registration, navigation and microscope-based AR support as well as two ultrasound systems (Flex Focus 800, BK5000, BK Medical, Herlev, Denmark), that are digitally fully integrated into the navigation systems (Ultrasound Navigation, Brainlab, Munich, Germany), allowing for a real-time overlay of iUS data onto navigation data sets as well as the intraoperative acquisition of iUS data sets that can be utilized for further navigational use.

Both ultrasound systems are equipped with comparable cranial transducers (Flex Focus 800: transducer 8862, BK5000: transducer N13C5, BK Medical, Herlev, Denmark), which are sterilizable and therefore can be used without an additional sterile cover that might impact image quality. In addition, using a sterile transducer rather than a non-sterile transducer covered with a sterile drape lowers the risk of loss of sterility during preparation and handling. The high-resolution N13C5 cranial transducer has a convex 29 mm × 10 mm contact surface and a scanning frequency of 5 to 13 MHz; the 8862 cranial transducer has the same spatial configuration but a scanning frequency of 3.8 to 10 MHz. Both transducers can be equipped with a sterile adapter and dedicated reference array with three reflective markers for navigated iUS usage after initial integration and technical calibration before the first overall use.

During navigated use, the probe is tracked in the patient coordinate system, allowing for an automatic real-time overlay of iUS data onto preoperative image data. For the acquisition of a 3D iUS data set, the transducer is constantly swept across the accessible field, and single 2D iUS images (with their corresponding position in the patient coordinate system) are acquired, which are then automatically stacked and post-processed by the navigation system (Ultrasound Navigation, Brainlab, Munich, Germany), resulting in a 3D iUS data set that can be further used during surgery.

### 2.3. Preoperative Planning

For surgical planning, MRI data acquired within a couple of days before surgery or resulting from routine diagnostics was used in all cases, typically including a contrast-enhanced T1-weighted 3D data set. Partially T2-weighted MRI data and/or time-of-flight MRI angiography (ToF) data were included. On the day before surgery, at least seven self-adhesive skin markers were attached to the patient’s head, and preoperative computed tomography (CT) imaging was performed to allow for a fiducial-based intraoperative patient registration procedure. 

After rigid image registration of all included data sets using the Image Fusion Element (Brainlab, Munich, Germany), the lesion as the target structure, as well as the transverse sinuses and risk structures were outlined manually using the Smart Brush Element (Brainlab, Munich, Germany). In addition, depending on its spatial relation to the lesion, the brainstem was automatically delineated using the Anatomical Mapping Element (Brainlab, Munich, Germany), especially in case of close spatial relation to the lesion, and partially reshaped to further fit the individual patient image data. Case dependent, further additional structures such as vessels in close vicinity to the lesion were outlined manually.

### 2.4. Patient Positioning and Registration Procedure

All patients underwent navigation-assisted surgery in a semi-sitting position. Therefore, after induction of anesthesia and implementation of venous air embolism (VAE) monitoring (esophageal ultrasound probe) and insertion of a catheter placed in the right atrium of the heart to aspire air bubbles in case of embolism, the sitting position was achieved in a stepwise manner, by gradually bending and tilting the operating table until an angle of less than or equal to 90 degrees between the trunk and inferior limbs with a suitable knee flexion was obtained. The ankle joints were leveled in line with the atrium to achieve venous counterpressure. The patient’s head was fixed in a metallic 3-pin head clamp (DORO^®^ QR3 Cranial Stabilization System, Black Forest Medical Group, Freiburg im Breisgau, Germany) adapted to the operating table. 

For navigation purposes, a patient reference array with four reflective markers was attached on the right side of the head clamp close to the surgical field to increase navigation accuracy with direct line-of-sight of the navigation system’s stereo camera (Curve Navigation, Brainlab, Munich, Germany). Fiducial-based patient registration was performed by matching the attached self-adhesive skin markers with the virtual corresponding markers in the preoperatively acquired data set to enable neuronavigation-supported surgery, and registration quality was verified. Afterwards, the non-sterile reference array was removed, followed by skin disinfection and sterile draping, and a sterile reference array was attached. Pointer-based navigation, as well as microscope-based AR navigation support, was enabled in this way. 

### 2.5. Intraoperative Navigated Ultrasound

Depending on the availability of a sterile transducer, preferably the BK5000, or the Flex Focus 800 (BK Medical, Herlev, Denmark) ultrasound system, with the corresponding cranial transducers, N13C5 (BK5000) or 8862 (Flex Focus 800), equipped with a reference array was utilized. The penetration depth was standardized to 65 mm (N13C5) or 62 mm (8862), respectively. To ensure high technical accuracy of the navigated transducer, contributing to overall accuracy, the calibration quality of the navigated transducer was visually inspected using a dedicated iUS phantom with integrated wires. Therefore, the concordance of calculated and within the ultrasound visualized wire crossings are visually verified to ensure high technical accuracy.

In supratentorial applications for iUS usage, the patient’s head is typically positioned in a way that at least a thin coupling fluid depot could be built up. In the sitting position, no stable saline depot can be built up. Therefore, continuous saline application close to the transducer is necessary while gently moving the probe across the dura to limit artifacts and generate interpretable iUS data sets. 

Intraoperative navigated ultrasound was performed in all cases before dural opening. After exploration of the surgical field using the navigated ultrasound probe to evaluate the overall applicability in each case, which might be hampered due to dural artifacts, especially in repeated surgery, in case of non-echogenic lesions or lesions close to bony structures, at least one 3D iUS data set was acquired with a high sampling rate and mean slice thickness of 0.5 mm. Therefore, the navigated probe was constantly swept and moved across the accessible dural area in the cranio-caudal direction to acquire an almost axial data set. Depending on accessibility and limited line-of-sight issues, a second nearly sagittal data set was in part acquired in the left–right direction. 

Real-time navigated iUS overlaid onto the preoperative MRI data, as well as the acquired 3D iUS data set (reference array-based co-registration), were then intraoperative visually inspected according to navigation accuracy. If navigation accuracy was rated sufficient by the surgeon, surgery was continued using MRI and iUS data in parallel, whereas in case navigation accuracy was rated insufficient, the lesion was manually outlined within the newly acquired 3D iUS data set, MRI data were discarded, and iUS-based navigation and microscope-based AR support including the iUS-based tumor outlines were further used intraoperatively. 

### 2.6. Additional Postprocessing Using Rigid Image-Based Co-Registration

Retrospectively, additionally, rigid image-based co-registration of iUS and MRI data was used, provided by the SNAP Element (Brainlab, Munich, Germany), released during the course of this study. This rigid image-based co-registration relies on previously published work by Wein et al. [27,28], applying a Linear Correlation of Linear Combination (LC2) similarity metric, allowing for rigid image-based MRI-iUS co-registration in a matter of seconds. SNAP is now fully integrated into the navigation system in addition to the standard reference array-based registration of the iUS data set implemented in the clinical standard navigation workflow. 

To evaluate the potential of rigid image-based co-registration in relation to reference array-based co-registration, potentially also enabling the integration of all preoperative generated information into the intraoperative data set, all iUS data sets were additionally rigidly co-registered with the preoperative MRI data sets using this rigid image-based co-registration approach implemented via SNAP. Therefore, within the Image Fusion Element (Brainlab, Munich, Germany), the MRI-iUS image pair is selected, initially still aligned according to the reference array-based co-registration, and then rigid image-based co-registration is initiated, without any further manual refinement. 

### 2.7. Quantification of Navigation Accuracy

Navigation accuracy was estimated using different approaches. 

First, intraoperatively, the visual matching of automatically overlaid preoperative MRI-based tumor outlines or outlines of other segmented structures on navigated iUS data was evaluated. If the surgeon decided on a “sufficient match”, MRI-based navigation was used as initially intended. In the case of a severe mismatch, iUS-based navigation was used further during surgery with intraoperative tumor segmentation based on the navigated 3D iUS data set. Second, postoperatively, navigation accuracy was analyzed in all cases using tumor outlines based on preoperative MRI data as well as tumor outlines based on intraoperatively acquired 3D iUS data sets. Manual segmentation was performed by two experts (M.B., A.G.), and the intraclass correlation coefficient (ICC) was calculated to ensure reliability of the segmentation process. Third, depending on the available data sets, postoperatively, up to ten uniquely identifiable landmarks (LMs) were manually defined within the preoperative MRI and the intraoperative 3D iUS data sets (M.B.). All landmarks were chosen based on clear visibility in both modalities, encompassing different tissue classes (e.g., gyri, sulci, vessels, tentorium) and various distances to the lesion, depending on the acquired iUS volume. All landmarks were technically verified to ensure correct labeling of the base data set and were reviewed by a second expert (A.G.) to ensure high quality.

Navigation accuracy was then assessed following a lesion-based analysis utilizing the Dice coefficient as a measure of spatial overlap and a landmark-based analysis using the overall Euclidean distance between corresponding landmarks for the standard reference array-based registration and the rigid image-based co-registration.

The Dice coefficient (*DSC*) [29] is a widely used parameter in medical imaging studies to quantify the degree of spatial overlap between two outlined objects, in this case, derived from MRI and iUS data, with poor agreement (*DSC* < 0.2), fair agreement (0.2 ≤ *DSC* < 0.4), moderate agreement (0.4 ≤ *DSC* < 0.6), good agreement (0.6 ≤ *DSC* < 0.8), and excellent agreement (0.8 ≤ *DSC* ≤ 1.0) [29], and is calculated as follows:

$$DSC\left({object}_{MRI},{object}_{iUS}\right)=\frac{2\times volume({object}_{MRI}\cap {object}_{iUS})}{volume\left({object}_{MRI}\right)+volume\left({object}_{iUS}\right)}$$

The mean Euclidean distance (*ED*) between corresponding landmarks per case was calculated for the reference array as well as for rigid image-based co-registration as a measure of navigation accuracy as follows:

$$ED\left({LM}_{MRI},{LM}_{iUS}\right)=\frac{1}{n}\sum_{k\in {LM}_{MRI},l\in {LM}_{iUS}}^{n}\sqrt{{\left(x_{k}-x_{l}\right)}^{2}+{\left(y_{k}-y_{l}\right)}^{2}+{\left(z_{k}-z_{l}\right)}^{2}}$$

### 2.8. Statistical Analysis

Statistical analysis was performed using the open-source software jamovi from the jamovi project (Version 2.3.21, computer software, retrieved from https://www.jamovi.org, accessed on 20 March 2024) [30]. The Shapiro–Wilk test was used to test for normal distribution of differences between groups as a prerequisite for the paired *t*-test. If no normal distribution was given, the Wilcoxon signed-rank test was applied as a non-parametric test. A two-way random ICC model was used to calculate the absolute agreement of manual segmentations. The significance level was set to *p* < 0.05.

## 3. Results

### 3.1. Patient Characteristics

In total, 15 patients (male/female: 6/9, mean age: 60.27 ± 9.33 years) were included in this study. Twelve patients underwent surgery for one, two patients for two, and one patient for four cerebellar lesions in the semi-sitting position. Neuropathological diagnosis revealed metastasis (n = 8, including three patients with multiple lesions), glioma (n = 2), meningioma (n = 1), subependymoma (n = 1), cavernoma (n = 1), hematoma (n = 1), and arteriovenous malformation (n = 1). In 14 cases, an osteoclastic approach (with/without C1 laminectomy: 12/2), and in one case, an osteoplastic suboccipital (midline/midline-right: 13/2) approach was chosen. In three cases (20.00%), intraoperatively, air bubbles were detected by transiently transesophageal echocardiography (TEE), which could be promptly and successfully treated. In the remaining twelve cases (80.00%), no VAE-related events or intraoperative complications were recorded; for further details, see Table 1.

### 3.2. Tumor Characteristics and Navigation Accuracy

In total, 20 lesions in 15 patients were analyzed. Table 2 summarizes the tumor volumes segmented within the preoperative MRI and intraoperative US data sets. Manual segmentation by both experts led to an ICC of 0.994 (MRI) and 0.968 (iUS), showing excellent agreement. The manual segmentation of the tumor outlines based on preoperative MRI data revealed a mean tumor volume of 9.88 ± 10.61 cm^3^ (min: 0.08 cm^3^, max: 30.50 cm^3^), and based on the iUS data, the mean tumor volume was 9.15 ± 9.68 cm^3^ (min: 0.08 cm^3^, max: 29.50 cm^3^). The statistical analysis using the Wilcoxon signed-rank test (no normal distribution given according to Shapiro–Wilk test, *p* < 0.001) revealed no significant differences in tumor volumes gained by MRI-based and iUS-based tumor segmentation (*p* = 0.355).

**Figure 1:**
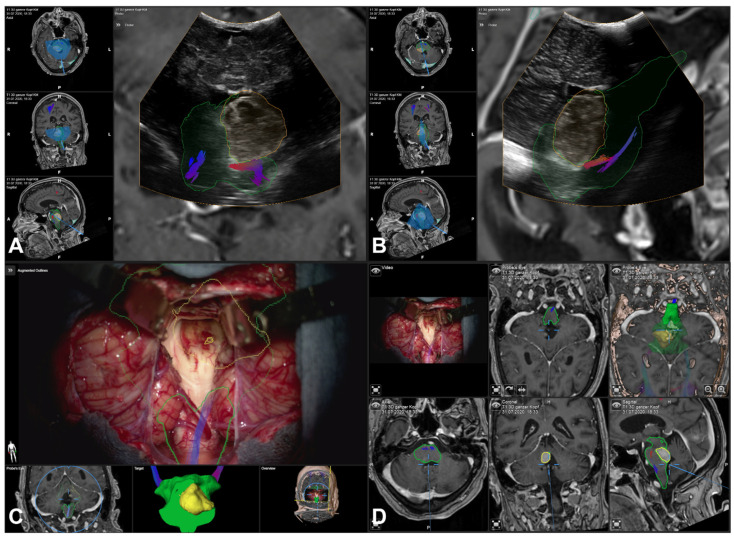
Navigated intraoperative ultrasound (transducer 8862) revealed a “sufficient” navigation accuracy, as shown by the outlined contours of the MRI-based information (yellow/orange) overlaid on the iUS data set in estimated axial (**A**) and sagittal (**B**) slicing, also including outlines of the brainstem (green) as well as fiber tractography of the corticospinal tract. Showing no need for a navigation update, MRI-based preoperative information (tumor, brainstem, and corticospinal tract) was used throughout the surgery for microscope-based AR (**C**) and overall navigation support (**D**).

**Figure 2:**
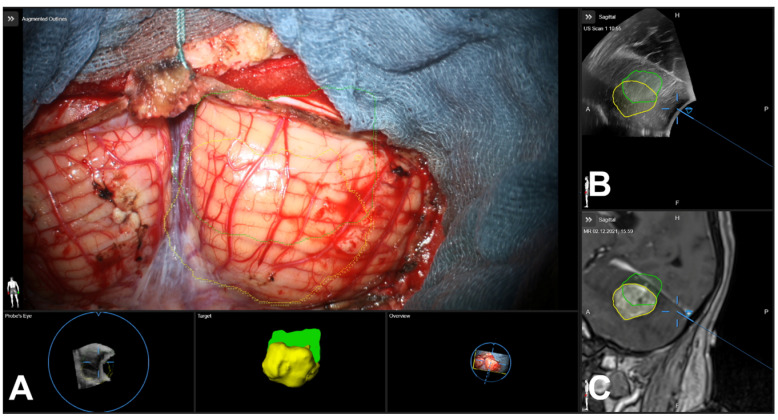
Navigated intraoperative ultrasound (transducer N13C5) revealed an “insufficient” navigation accuracy, as shown by the outlined contours of MRI-based information (yellow) and iUS-based segmentation (green) of the lesion within the microscope-based AR view (**A**), the iUS (**B**), and the MRI data set (**C**). Following this rating, during surgery, only the iUS data and iUS-based segmentation were used for navigational purposes.

Intraoperatively, relying on reference array-based registration, in seven cases, the navigation accuracy was rated as “sufficient” (see Figure 1) and in the remaining eight cases was “insufficient”. In the latter cases, the lesion was outlined manually within the acquired iUS data set. Surgery was then continued with iUS-based navigation and microscope-based AR utilizing the new outlines (see Figure 2). 

### 3.3. Lesion-Based Analysis

Regarding reference array-based registration, the mean Dice coefficient comparing the spatial overlap between the MRI- and iUS-based tumor segmentation was 0.42 ± 0.30, ranging from 0.00 to 0.87. The spatial overlap was rated as “poor” for five, “fair” for five, “moderate” for three, “good” for five, and “excellent” for two lesions. For the rigid image-based registration of the preoperative MRI and intraoperatively acquired US data based on tumor outlines, a mean Dice coefficient of 0.65 ± 0.23 was calculated, ranging from 0.00 to 0.88. The spatial overlap was rated as “poor” for one, “fair” for one, “moderate” for four, “good” for eight, and “excellent” for six lesions. 

**Figure 3:**
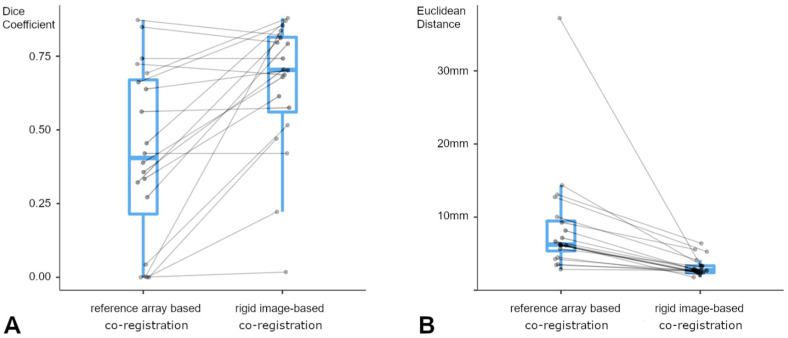
Plotted Dice coefficients (**A**) for reference array-based co-registration (left) and rigid image-based co-registration (right) show a significant improvement of spatial overlap between MRI- and iUS-based segmentation (paired *t*-test, *p* < 0.001). Despite its size dependency, in one case of a small lesion and low-volume iUS data set, spatial overlap did not improve significantly. Plotted mean Euclidean distances (**B**) for reference array-based co-registration (left) and rigid image-based co-registration (right) display a significant decrease (Wilcoxon signed-rank test, *p* < 0.001), even in a case with an initial mean offset of 37.60 mm.

Comparing the spatial overlap achieved by standard reference array-based registration and rigid image-based registration of preoperative MRI and intraoperative US, a significant increase (paired *t*-test, *p* < 0.001) in the spatial overlap was seen in favor of rigid image-based registration; see Figure 3A.

### 3.4. Landmark-Based Analysis

For standard reference array-based registration, the mean Euclidean distance as a measure for the registration accuracy between corresponding landmarks in the preoperative MRI data and intraoperative US data was 8.69 ± 6.23 mm (median 6.24 mm), ranging from 2.85 mm to 37.2 mm. For rigid image-based registration, the resulting mean Euclidean distance was 3.19 ± 2.73 mm (median 2.73 mm) with a minimum distance of 1.77 mm and a maximum distance of 6.40 mm. 

Comparing the mean Euclidean distance per lesion between the reference array- and image-based MRI-iUS registration as a measure of the registration quality, the image-based registration showed significantly lower distances (Wilcoxon signed-rank test, *p* < 0.001) between the corresponding landmarks and, therefore, a higher registration accuracy between the MRI and iUS data; see Figure 3B. 

### 3.5. Illustrative Cases

**Figure 4:**
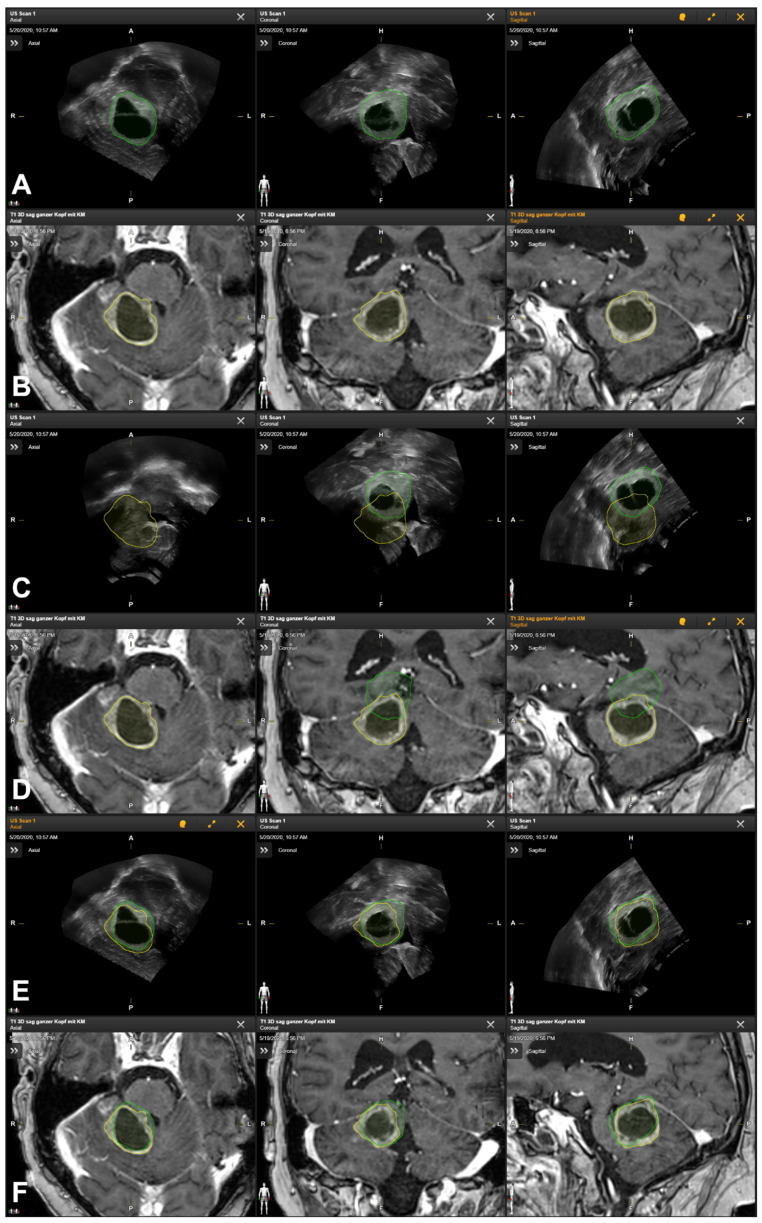
Manual segmentation of tumor outlines based on preoperative MRI data (segmentation in yellow, (**A**)) and navigated intraoperative US data (segmentation in green, (**B**)) showing the spatial mismatch of preoperative MRI-based tumor outlines and intraoperative iUS data leading to an iUS-based navigation update by manually outlining the tumor within the intraoperatively acquired US data set further used for navigation purposes in case of reference array-based registration (**C**,**D**) as well as good match between both data sets after rigid image-based registration of MRI and iUS data (**E**,**F**).

Patient No. 1 is a 42-year-old male patient with a cerebellar anaplastic astrocytic tumor. A suboccipital osteoclastic trepanation with C1 laminectomy was performed. Patient registration was achieved using a landmark-based registration with median initial accuracy. According to the intraoperatively acquired ultrasound data (178 slices, an average slice thickness of 0.5 mm), the lesion was clearly depictable and completely included in the data set. In parallel, a clinically significant mismatch between the preoperative MRI data and the MRI-based tumor segmentation (see Figure 4A) and the anatomical landmarks and tumor outlines within the iUS data set (see Figure 4B) was seen (see Figure 4C,D). Intraoperatively, the lesion was outlined manually within the iUS data set, and standalone iUS-based navigation support was used throughout the surgery with the outlined tumor also provided within the microscope-based AR navigation. 

Retrospective rigid image-based MRI-iUS co-registration (see Figure 4E,F) led to significantly increased co-registration results, improving the spatial overlap of the outlined lesion from 0.27 to 0.79. Assessing the accuracy based on ten uniquely identifiable landmarks, the mean Euclidean distance between the corresponding landmarks was 14.37 ± 1.97 mm (range: 10.76 mm to 17.31 mm), which was also improved to a mean Euclidean distance of 3.35 ± 1.43 mm (range 1.05 mm to 5.56 mm) for the image-based MRI-iUS registration.

**Figure 5:**
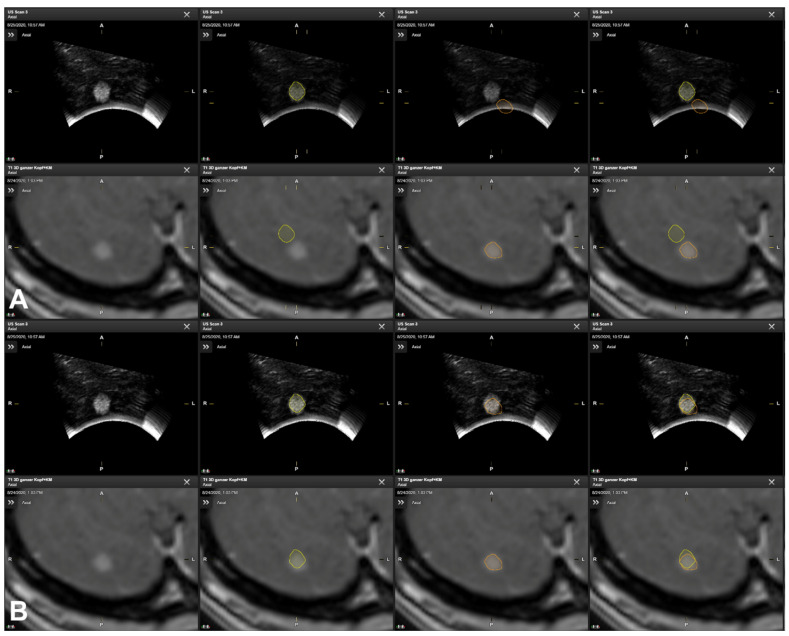
Manual segmentation of tumor outlines based on preoperative MRI data (orange) and navigated iUS data (yellow) showing the spatial mismatch seen in reference array-based co-registration, indicating the need for a navigation update (**A**), whereas navigation accuracy was increased after rigid image-based co-registration (**B**), showing a suitable spatial overlap of MRI- and iUS-based tumor outlines.

Patient No. 3 is a 62-year-old female patient with two colon carcinoma metastases in the cerebellum. The tumor volume was 8.54 cm^3^ and 0.09 cm^3^, respectively. A suboccipital osteoclastic trepanation with C1 laminectomy was performed. Patient registration was achieved using landmark-based registration with a median initial accuracy. In the first intraoperatively acquired ultrasound data set (125 slices, an average slice thickness of 0.5 mm), the larger lesion was clearly depictable and completely included, showing a “good” spatial overlap of the lesion outlines (*DSC* 0.74) with a moderate mean Euclidean distance of 4.5 mm. Further intraoperatively acquired ultrasound data (36 slices, an average slice thickness of 0.5 mm) covering the smaller lesion showed a clear discrepancy between the MRI and iUS data with no initial spatial overlap (*DSC* 0.00) of the outlined lesions and a mean Euclidean distance of 6.33 mm, which could be improved to a spatial overlap of 0.47 and a mean Euclidean distance of 1.77 mm, respectively, allowing for the intraoperative localization and identification of the lesion (see Figure 5). Intraoperatively, the smaller lesion was outlined manually in the iUS data set, and standalone iUS-based navigation support was used to continue navigation-supported surgery for this case.

## 4. Discussion

Since its introduction in the 1990s, neuronavigation, first used as pointer-based navigation and, later, also used in terms of microscope-based AR navigation, has proven its clinical benefits and usefulness and has become an indispensable and routine tool for a wide variety of neurosurgical applications in cranial and spinal surgical procedures [1,2,3,4,31]. Neuronavigation is known to support intraoperative surgical orientation, the precise planning of surgical trajectories, the identification of the spatial relationship of the lesion and functional risk structures (e.g., eloquent areas, fiber tracts, vascular structures), and contributes to pre- and intraoperative surgical decision making and extending radical resection with in-parallel increasing patient safety [4,32,33,34,35,36].

However, the benefits and surgical advantages of neuronavigation, also extended by microscope-based AR, easing the mental transfer of image data onto the surgical field, and its application safety fundamentally depend on the highly precise and accurate mapping of preoperatively acquired data on the intraoperative surgical situs—linking image space and physical space—at the beginning and throughout the entire course of surgery, which remains a multifactorial and essential challenge [1,2,4,15,32,35,37,38,39,40]. 

Besides non-user-dependent factors such as the intrinsic accuracy of the neuronavigation system itself, several factors can be addressed to increase the overall navigation accuracy. The overall navigation accuracy can be divided into the imaging, technical, and registration accuracy, the latter being the most prominent relevant contributing factor before intracranial manipulation when mapping the image and patient space. The intraoperative accuracy is mainly impaired by brain deformations during surgery due to the loss of CSF, increased swelling, the insertion of brain retractors, and the effects of gravity [4,15,16,33,34]. The navigation accuracy, in terms of an altered spatial relationship between the patient’s head and the reference array, intraoperatively, not yet hampered by the effects of brain shift, is known to decrease throughout surgery [3,41,42] and can be compensated for up to a certain point, e.g., by re-registration utilizing acquired landmarks [42] or in-plane adaptions of AR when using microscope-based AR navigation [15]. Non-linear deformations remain a fundamental challenge. Whereas a high clinical accuracy is expected throughout surgery, the clinical accuracy steadily decreases and can, in the worst case, lead to a total loss of navigation capabilities [3,4,43]. 

Being aware of this, neuronavigation support is commonly implemented in supratentorial neurosurgical procedures. However, it has not yet found its way into posterior fossa surgery in the sitting position due to severe concerns about impaired accuracy [11]. The prone and sitting positions are both commonly used for posterior fossa surgery [44], both providing advantages and challenges for neurosurgeons and neuroanesthesiologists; however, which approach to choose is still a controversial issue and needs to balance risks related to the positioning and surgical and anesthesiological advantages [12,13,44]. The prone position is a widely used patient positioning for posterior approaches, also shown to decrease the VAE risk compared to the sitting position. However, VAE is still reported to occur in 10% to 17% of all craniotomy cases in the prone position and is not exclusively seen in the sitting approach [12,44]. However, this positioning is also challenging under anesthesiological considerations in terms of providing and ensuring adequate oxygenation and ventilation, maintaining hemodynamics, and securing intravenous lines and the tracheal tube, especially the access to the patient’s airways. The sitting position offers a broad range of surgical advantages, such as the gravitational drainage of venous blood and CSF from the surgical site, improving surgical orientation, allowing for a cleaner dissection while reducing the need for bipolar coagulation, facilitating cerebellar retraction and access to midline structures and deep areas, a significant reduction in cerebellar swelling, cerebral venous decompression and reduced blood loss [12,13]. Anesthesiological benefits include ventilation with lower airway pressure, less impairment of diaphragmatic motion, improved access to the tracheal tube, and easier access to the patient in case of emergency [12,13,44]. However, there are also notable risks associated with this positioning technique. The two major reported complications include VAE with possibly paradoxical air embolism, which might be devastating in the presence of PFO, typically considered a contraindication, and intraoperative hypotension [12,13,44,45]. The VAE incidence is reported to be highly varying with an overall incidence of 39%, ranging from 1% to 76% [45], markedly depending on the monitoring method and settings used. TEE seems to be the most sensitive method, providing the early detection of even small VAEs of little clinical significance [12,45,46], as also seen in this study, with an incidence of 20%. In all the cases, only transient VAE-related events were recorded promptly and successfully treated.

The surgical advantages of the sitting position, e.g., the gravitational drainage of CSF, leading to non-linear deformations of the brain tissue in relation to the preoperative imaging data typically considered for neuronavigation support, lead to doubts about the applicability of neuronavigation assistance in certain cases [11]. However, in general, as previously shown, the intraoperative patient positioning differing from the preoperative patient positioning during MRI acquisition, as is the case not only in sitting but also in prone positioning, leads to inaccuracies in mapping images and patient space as early as during patient registration, which relevantly contributes to the overall navigation accuracy, and the mapping of intracranial structures [10,12,14]. Various approaches can be considered for patient registration, such as paired-point registration using anatomical, artificial, or bony landmarks, surface matching techniques, or automatic registration [3]. Registration errors throughout the manual registration process with high user-dependency arise due to skin distortions caused by gravity, shifting, reduction in muscle tonus, intubation, nasogastric tube placement, skin movement due to fixation within the head clamp, and skin shifting using the navigation pointer, and is highly user-dependent, but is also affected by varying patient positioning [14]. When using intraoperative imaging such as CT or MRI where available, automatic patient registration is a valuable and highly accurate registration method that is user-independent and independent of effects of patient positioning throughout the preoperative image acquisition, leading to mean target registration errors of less than 1 mm [1,47,48,49], but, however, is not utilizable in specific surgical positions such as the sitting position for accessing the posterior fossa [14]. Besides patient registration, the patient positioning differing between the preoperative MRI used for surgical planning and the intraoperative patient positioning also affects intracranial structures. Compared to the cerebrum, the posterior fossa includes a larger amount of CSF and larger subarachnoidal spaces, allowing for the increased movement of the brain depending on the patient’s position due to the effects of gravity and was reported, based on MRI studies, to vary up to 11.46 mm [10,50,51,52,53,54]. This supports previous findings of higher navigation inaccuracies in the prone and sitting position when targeting the posterior fossa with the partial loss of navigational support compared to standard supratentorial approaches [10,55,56].

However, discrepancies between the image and patient space throughout surgery are daily challenges in the application of neuronavigation, and there are several opportunities to compensate for them [15,16,31,34,39,57,58]. While rigid positional shifts, e.g., due to a change in the relation between the reference array and the patient’s head, can be accounted for by re-registration, inaccuracies caused by intraparenchymal non-linear shifting due to the positional effects of patient positioning and surgical manipulation (e.g., loss of CSF, swelling, retractors, gravity) over the course of surgery can be overcome using intraoperative imaging techniques or partially by AR [15]. While the usage of iMRI is generally limited due to its availability, its high cost and time consumption, and also the intraoperative patient positioning, such as the sitting position, limits its applicability [16,17]. First introduced in the 1980s, iUS gained attraction due to its capability for real-time imaging and repeatability without significant interruption of the surgical workflow. It is cost-efficient, safe, and straightforward to use [18,59]. Over the years, iUS technology has undergone tremendous advancements, and nowadays offers an improved spatial and priceless temporal resolution that overcomes the initial limitations caused by a comparably low resolution, high user dependency, and lack of training and experience [59]. Enabling oblique imaging, the anatomical orientation within the limited field of view of iUS data due to the spatial restrictions of the craniotomy is challenging, and users might be encouraged to use iUS approximately aligned along the conventional anatomical axis (axial, coronal, sagittal) to allow for more intuitive understanding [59]. With its integration into neuronavigation systems, tracking the transducer similarly to other navigation tools allows for the regular exploration of the navigation accuracy, brain shift, and extent of resection, depending on the echogenicity of the lesion. With advancements in 3D iUS imaging (3D transducer or stacking of 2D images), it even offers the opportunity to update navigation by usage of the intraoperatively acquired 3D iUS data, accurately representing the recent intraoperative configuration of the brain following patient positioning, brain shift, and so on. Allowing for its navigated use, iUS has gained more attraction and has become an essential part of surgical routines in many setups [19,20,21,22,23,24,25]; most sites follow a specific standardized protocol, which supports a steep learning curve that allows for the transfer of this technique from simple to complex cases through technical and clinical experience, and experience in the interpretation of iUS data and the visual matching of multimodal data for navigational purposes. IUS has shown a good correlation with iMRI in determining the extent of resection and the identification of tumor remnants in metastases and glioma surgery and, thus, especially in combination with navigation, facilitates tumor removal and extended resection, improving the overall patient survival and quality of life [60,61].

In this proof-of-concept study, the capability of navigated iUS to identify navigation inaccuracies and to compensate for those inaccuracies was evaluated. In doing so, iUS was conducted before the dural opening as part of our institutional routine but can also be repeated at any time after the dural opening and over the course of resection depending on the surgeon’s decision to verify and update navigation, to identify remaining tumor, or to determine the extent of resection intraoperatively. The initial patient registration was, in all cases, performed by landmark-based registration with medium to high accuracy, depending on the quality of the preoperative imaging, skin shift, application of adhesive skin fiducials, and user-dependent registration. Within the clinical workflow observed in this study, the registration of iUS and preoperative MRI images was based on the spatial information of the precalibrated iUS probe within the patient coordinate system (reference array-based registration) and thus provided an estimate of the concordance between the pre- and intraoperative data [25,62,63,64,65,66] and allowed for an estimate of the navigation accuracy encompassing the positional and intraparenchymal shift. Consequently, if a “severe” mismatch was seen, disabling the use of navigation support, navigation support including microscope-based AR was enabled again by outlining the lesion within the iUS data set and continuing surgery based on iUS alone, thereby losing MRI-based pre-segmented information about related structures. Keeping this information while using the “up-to-date” spatial information provided by the intraoperative ultrasound requires image-based co-registration approaches and the matching of pre- and intraoperative data to enable multimodal navigation support throughout the surgery. Various rigid image-based MRI–iUS co-registration methods are available in research setups [67,68] based on varying approaches such as feature extraction and descriptors or, e.g., the use of hyperechogenic structures and joint probabilities. Another approach implemented rigid co-registration for CT and iUS data [27] and was later adapted to MRI and iUS data implementing the Linear Correlation of Linear Combination (LC2) similarity metric, allowing for rigid image co-registration over a couple of seconds [28], which has been successfully technically evaluated within the CuRIOUS2018 Challenge [69]. Based on this approach, rigid image-based MRI-iUS co-registration has been implemented (Brainlab, Munich, Germany), and a prior release has been applied in a retrospective case series including patients with intracranial lesions in a comparable setup as that used in this study [70] and released as a fully integrated part of the navigational system, easing the intraoperative workflow and usage. The results of the CuRIOUS2018 Challenge and the prerelease evaluation of a prototype showed a significant decrease in the navigation inaccuracy by iUS-based navigation updates relying on rigid image-based co-registration. Contrary to the previous reports, in this study, on the one hand, a lesion-based similarity metric (Dice coefficient) as well as a landmark-based similarity metric (Euclidean distance) was assessed for the analysis of the navigation accuracy with respect to the reference array-based approach as well as to the rigid image-based MRI-iUS co-registration.

The statistical analysis of the tumor volumes outlined in the preoperative MRI and iUS data did not differ significantly, suggesting that both modalities can be considered comparable in terms of tumor delineation, being a prerequisite for the lesion-based analysis of navigation accuracy, which is also in line with previous studies showing the comparability of MRI and iUS in tumor delineation [21,25,71]. Therefore, the spatial overlap of the segmented tumor volumes was assessed using the Dice coefficient, ranging from 0.00 (no overlap) to 1.00 (perfect match), as a measure of the lesion-based co-registration quality. The reference array-based registration revealed a Dice coefficient of 0.42 ± 0.30, ranging from 0.00 to 0.87, whereas rigid image-based co-registration was significantly improved to 0.65 ± 0.23, ranging from 0.00 to 0.88. The Dice coefficient seen in reference array-based co-registration was lower than that in previous studies, including supratentorial lesions only [21,25], underpinning the potentially decreased navigation accuracy expected in the sitting position when assessing the posterior fossa. Despite small lesions having a higher likelihood of low spatial overlap due to the Dice coefficient’s dependency on object size [72,73], the significant increase in the spatial overlap relying on rigid image-based co-registration now offers the opportunity to perform iUS-based navigation updates even in cases of severe mismatch, as seen in one case with an increase in the spatial overlap from 0.00 to 0.84 (tumor volume 30.50 cm^3^), not only contributed to by brain shift but also by a positional shift most plausibly due to the handling of the reference array [3,15]. In the case of a smaller lesion, the acquired iUS data set used for co-registration might also only cover a small range (as partially seen in Table 2), limiting the anatomical information in the iUS data set besides the lesion itself that is employed within the rigid co-registration approach, and therefore potentially leading to a limited improvement concerning the spatial overlap. However, in the case of a small lesion with an iUS data set covering a larger portion of the brain, a higher improvement was seen. 

Besides the spatial overlap of lesions seen in the MRI and iUS data, the quality of co-registration was also assessed in a landmark-based manner, comparing the distances between the corresponding landmarks in both modalities, as previously performed in a recent pilot study with supratentorial lesions [70]. In this study on infratentorial lesions surgically treated in the sitting position, initial reference array-based registration led to a mean Euclidean distance of 8.69 ± 6.23 mm (ranging from 2.85 mm to 37.2 mm), which was also significantly improved by rigid image-based co-registration with a mean Euclidean distance of 3.19 ± 2.73 mm (ranging from 1.77 mm to 6.40 mm). Specifically, as seen in one case with an initial maximum Euclidean distance of 37.2 mm between the corresponding landmarks, not only the intraparenchymal shift but also the positional shift of the rigid structures (handling of, e.g., reference arrays) contributed to the overall high navigation inaccuracy in this specific case. The mean Euclidean distance between the corresponding landmarks based on reference array-based co-registration was therefore due to the configuration of the posterior fossa being expectedly higher than in previous studies with supratentorial lesions, even though studies on navigation within the posterior fossa and the explicit analysis of navigation accuracy are rare [11]. The main effects of brain shift are expected after dural opening. Still, larger discrepancies between MRI and iUS data have already been reported for applications in supratentorial lesions with mean distances of 3 mm to 4 mm even before dural opening. Higher inaccuracies might be caused by mechanical, operational, and technical effects (e.g., skin shift, shift of markers, forces during craniotomy, interchange of non-sterile/sterile reference array, draping) [2,3,15,52], the manual patient registration procedure, which is also reported to have a limited accuracy with a target registration error of about 1.8 to 5.0 mm [3,49,74], and intraparenchymal shift due to patient positioning varying from the position during preoperative imaging, especially within the posterior fossa [10,50,51,52,53,54]. However, after rigid image-based co-registration, the mean distance significantly decreased, also being in the range of the target registration error of the manual registration procedure itself; however, it is not close to zero yet. This is partially caused by some sort of uncertainty in detecting the same anatomical structures in both modalities and the choice of anatomical structures (hard/soft tissue landmarks) being shifted differently due to gravitational effects [11]. On the other hand, brain shift caused by gravitational effects even before dural opening; deformations caused by the convex iUS probe when gently pushing the probe on the dura while swiping across the accessible area in the craniotomy for improved image quality and coupling; and also in the later course of surgery, brain shift due to CSF loss results in non-linear deformations of the brain tissue that cannot be fully compensated for using only rigid co-registration approaches. However, there is a need for non-linear co-registration methods combining preoperative MRI and intraoperative US to overcome these limitations [59,75,76,77,78,79], as existing methods provide promising results but remain computationally time-consuming, limiting their intraoperative applicability. Also, the image quality seems to be a relevant factor that contributes to the overall co-registration quality. The iUS volume needs to cover a broad area of the brain tissue containing a lot of structural information for co-registration, which might be limited due to small craniotomies and requires small high-resolution footprint probes [59]. The typical acquisition of 3D iUS data sets using 2D probes is prone to acquisition errors and distortions while manually sweeping the probe across the situs, which might be overcome by the introduction of matrix phased array probes immediately generating a 3D volume without any sweep, but with a lower resolution [59,80].

Although neuronavigation support is routinely implemented in neurosurgical procedures, including microscope-based AR and intraoperative imaging, doubts about its applicability in the sitting position when targeting the posterior fossa limit its applicability even though it is imperative to choose the best surgical approach and optimal trajectory while limiting tissue dissection to a minimum [11]. This is also the case for supratentorial lesions, especially in case of small lesions or lesions that are difficult to identify in situ. The use of navigated iUS offers a straightforward opportunity to verify the navigation accuracy and, thus, to confirm its applicability or to update navigation by either using the iUS data set itself as the basis for navigation support or by adapting the preoperative planning data to the iUS data set, overcoming the limitations of accuracy and fully benefitting from intraoperative navigation support for maximum safe tumor resection. Specifically, in cases where lesions might not be clearly identifiable in iUS data or risk structures in close vicinity of the lesion, the co-registration of preoperative and intraoperative data offers a unique chance for multimodal navigation support throughout surgery, as is also routinely used in other cranial approaches. Navigation support might also assist in the education and training of residents and less experienced surgeons, supporting intraoperative orientation and allowing for the mental mapping of the surgical trajectory, the surgical field, intraoperative landmarks, and multimodal image data while harnessing the surgical advantages of the sitting position.

The limitations of this study encompass its retrospective character and the small sample size due to the stringent inclusion and exclusion criteria (e.g., single surgeon, lesion fully visible, no cerebellopontine angle tumors) aiming to reduce the variability in this proof-of-concept study. Having proven the applicability in this small and “optimal” cohort, this needs to be transferred to other cases with non-optimal image data with respect to lesion coverage, iUS volume, varying image quality, and different users to underpin its general usability. Due to its retrospective character and the availability of the recently released rigid image-based co-registration approach, which was not considered intraoperatively in this study, the immediate intraoperative clinical comparison of both methods cannot be performed yet, and further studies are needed to evaluate its clinical intraoperative benefits (subjective to the surgeon). Therefore, prospective studies should also include iUS data acquisition before and after dural opening and during surgery to analyze and support the applicability of iUS-based navigation updates in the later course of surgery. 

The loss of navigation accuracy and brain shift are common challenges in neurosurgical applications, and iUS offers an opportunity to analyze these. The biomechanical properties of the brain tissue, as well as brain shift dynamics depending on different surgical positions, could also be further investigated, allowing for a deeper understanding of the navigation applicability and intraoperative needs for navigation updates.

## 5. Conclusions

Navigated intraoperative ultrasound can serve as an ideal and easy-to-use tool to enable navigation and microscope-based AR support in posterior fossa surgery in the sitting position, offering various surgical benefits but also being associated with a higher risk of navigation inaccuracies. Navigated iUS therefore provides real-time information about the navigation accuracy and an opportunity to update navigation support according to the recent intraoperative challenges of positional and intraparenchymal alternations and non-linear deformations of the brain and, in this way, supports the intraoperative orientation and allows for the mental mapping of the surgical trajectory, surgical field, landmarks, and multimodal imaging data, not only for experienced surgeons but also for younger residents and less experienced surgeons. 

## Figures and Tables

**Table 1:** Patient characteristics.

Patient No.	Age	Sex	Diagnosis
1	42.70	M	Anaplastic astrocytic tumor
2	76.56	F	Hematoma (brainstem)
3	71.00	F	Metastasis (colon) ^1^
4	62.04	M	Metastasis (lung) ^2^
5	67.55	F	Metastasis (neuroendocrine carcinoma)
6	53.91	F	Metastasis (ovary)
7	61.53	M	Metastasis (colon)
8	63.08	F	Meningioma
9	45.00	F	Low-grade glioma
10	59.93	M	Metastasis (gastrointestinal)
11	59.43	M	Metastasis (colon)
12	68.71	F	Metastasis (ovary) ^1^
13	64.91	M	Cavernoma
14	51.08	F	Subependymoma, WHO°I
15	56.60	F	Arteriovenous malformation

^1^ Two lesions, ^2^ four lesions.

**Table 2:** Tumor object and iUS characteristics.

Patient No.	Tumor Volume MRI [cm³]	Tumor Volume iUS [cm^3^]	iUS Probe	iUS #Slices/Slice Thickness
1	13.10	13.40	N13C5	178/0.5 mm
2	5.40	5.55	8862	98/0.5 mm
3	8.54	8.60	8862	125/0.5 mm
	0.09	0.08		36/0.5 mm
4	4.45	3.76	N13C5	155/0.5 mm
	0.08	0.10		
	0.45	0.48		
	2.44	2.27		
5	30.50	26.40	8862	175/0.5 mm
6	10.50	10.40	N13C5	110/0.5 mm
7	22.10	21.80	N13C5	120/0.5 mm
8	21.30	21.30	N13C5	128/0.5 mm
9	0.43	0.51	8862	81/0.4 mm
10	29.40	29.50	N13C5	176/0.4 mm
11	18.90	14.60	N13C5	101/0.5 mm
12	24.00	17.80	N13C5	206/0.5 mm
	0.60	0.52		60/0.5 mm
13	13.10	13.40	N13C5	134/0.5 mm
14	5.40	5.55	N13C5	76/0.4 mm
15	8.54	8.60	N13C5	159/0.5 mm

## Data Availability

The data in this study are available on request from the corresponding author. The data are not publicly available due to privacy restrictions.
